# Are Left- and Right-Eye Pupil Sizes Always Equal?

**DOI:** 10.16910/jemr.12.2.1

**Published:** 2019-07-02

**Authors:** Nergiz Ercil Cagiltay, Gonca Gokce Menekse Dalveren

**Affiliations:** Atilim University, Faculty of Engineering, Department of Software Engineering, Ankara, Turkey; Norwegian University of Science and Technology, Department of Computer Science, Gjøvik, Norway; Atilim University, Faculty of Engineering, Department of Information Systems Engineering, Ankara, Turkey

**Keywords:** Left-eye and right-eye pupil size, haptic control, intermediate and novice surgical residents, endo-neurosurgery

## Abstract

Eye movements provide very critical information about the cognitive load and behaviors of human beings. Earlier studies report that under normal conditions, the left- and right-eye pupil sizes are equal. For this reason, most studies undertaking eye-movement analysis are conducted by only considering the pupil size of a single eye or taking the average size of both eye pupils. This study attempts to offer a better understanding concerning whether there are any differences between the left- and right-eye pupil sizes of the right-handed surgical residents while performing surgical tasks in a computer-based simulation environment under different conditions (left-hand, right-hand and both hands). According to the results, in many cases, the right-eye pupil sizes of the participants were larger than their left-eye pupil sizes while performing the tasks under right-hand and both hands conditions. However, no significant difference was found in relation to the tasks performed under left-hand condition in all scenarios. These results are very critical to shed further light on the cognitive load of the surgical residents by analyzing their left-eye and right-eye pupil sizes. Further research is required to investigate the effect of the difficulty level of each scenario, its appropriateness with the skill level of the participants, and handedness on the differences between the leftand right-eye pupil sizes.

## Introduction

Today, with the technological improvements, it has become possible to measure the pupil size of individuals. This provides important data to offer a better understanding of their cognitive processing while performing different tasks. Earlier studies reported a close relationship between pupil size and cognitive load. It has been suggested that an increase in pupil size is an indicator of an increase in the cognitive load (1, 2, 3, 4, 5,6, 7, 8). For instance, Zheng, Jiang (9) found that in laparoscopic procedures, the subjects’ peak pupil sizes increased in parallel to the increase in the task difficulty levels. Other studies also showed the presence of a relation between attention and eye movements (10, 11). According to an earlier study in which surgical tasks were performed under different hand conditions (both hands, dominant hand or non-dominant hand), there were changes in the pupil sizes of the surgical residents, indicating that when tasks were performed under the both-hands condition, they were considered more difficult than the dominant and non-dominant hand conditions (12).

Furthermore, several studies reported that there were differences in brain activities and cognitive strategies of experts and novices for skill-based tasks (13, 14, 15, 16, 17). For instance, Foroughi, Sibley (18) determined that trial completion times and maximum pupil size were significantly reduced across trials, indicating that through pupil size information about the individuals’ level of learning changed from the learned procedure state to an automatic processing of information when learning a new task.

Hence, as an objective measure, today pupil size offers several insights into the cognitive load of human beings performing certain tasks (19). Blumenfeld (20) reported that under normal conditions, the left- and right-eye pupil sizes were equal. For this reason, studies investigating the pupil size are usually conducted by either measuring a single eye (4, 18, 21) or taking the average pupil size of both eyes (7, 9, 12, 22, 23). However, there are studies reporting that in the dominant hemisphere, the expansion of the hand motor cortex may provide an extra space for the cortical encoding of a greater motor skill repertoire of the preferred hand, which indicates that for the right-handed people, the left hemisphere is dominant for manual skills (24). Additionally, researchers showed that patterns of activity within the premotor and posterior parietal cortex vary systematically according to the specific type of hand action being imagined (25). Besides, it is known that the task involved the right hand movements causes a unilateral activation of the left central sulcus in the hand region of the motor homunculus (26).

In brief, even it is accepted as an important measure, the left- and right-eye pupil sizes of individuals have not yet been compared under different conditions. In the literature, there are very limited number of studies considering the differences in pupil sizes between the left and right eye. As summarized in the study of Wahn, Ferris (27), the structures related to pupil size control might be systematically affected with the right-lateralization of attentional processing, causing differences in the left and right pupil sizes. Studies suggested that the differences in the left- and right- eye pupil sizes might be associated with attentional processing (28). Wahn, Ferris (27) similarly reported that an increase in the task experience uncovered modulations in pupil size asymmetries (left- and right-eye pupil size differences). These results all indicate the presence of a relation between experience, cognitive load, and changes in the left and right pupil sizes, but this has not yet been investigated in detail in the literature.

Accordingly, under different hand conditions (right-hand, left-hand and both hands), this study attempts to provide a better understanding of the changes in the left and right pupil sizes of novice and intermediate level endo-neurosurgery residents. The main assumption of this study is that even the left and right pupil sizes are equal under normal conditions (20), and under different hand conditions, some differences may be observed when performing endo-neurosurgery tasks. Revealing these differences will help demonstrate the closer connections between the cognitive load and skill-based task performance. This information can also elucidate the behaviors of groups with different skill levels.

## Methods

In order to fully grasp the influence of the experience level and hand condition on the mental workload of surgical residents, the right-eye and left-eye pupil sizes of 20 right-handed participants were examined using computer-based simulated surgical tasks included in four scenarios, namely catch, reach, clean, and follow.

### Participants

Twenty participants from ear-nose-throat (ENT) surgery and neurosurgery departments of a medical school voluntarily participated in this study. Most of the participants were male (90%) and did not use prescription glasses (70%) (Table 1). In a specific field such as ear-nose-throat (ENT) surgery and neurosurgery it is very difficult to encourage surgeons to participate, therefore, researches in this field usually conducted with limited number of participants (29, 30, 31).

**Table 1:** Participant Descriptive Information

		n	%
Gender	Female	2	10
	Male	18	90
Prescription Glasses	No	14	70
	Yes	6	30
Surgical Experience	Novice	11	55
	Intermediate	9	45

In their study, investigating the expertise and skill levels of surgeons in minimally invasive surgery procedures, Silvennoinen, Mecklin (32) defined novices as those who had gained basic knowledge of minimal invasive surgery, and intermediate surgeons as those who had started to perform minimal invasive operations (32). According to this description, of the 20 participants in the current study, 11 were novices with an average age of 28.45 (SD = 7.69) years, who worked as a research assistant in the ENT or neurosurgery departments (Table 2). None of the participants had previously performed endoscopic surgery on their own. The novice participants had observed 11.18 (SD = 14.23) and assisted 4.54 (SD = 11.37) surgical procedures. Nine participants were intermediates with an average age of 29.33 (SD = 1.50) years, and they had observed 48.33 (SD = 31.62), assisted 32.00 (SD = 24.19) and performed 16.56 (SD = 16.60) surgical procedures as surgeons. All the participants were right-handed.

**Table 2:** Participant Endoscopic Surgery Experience

Participant	Age	Monitored	Assisted	Performed
Intermediate	29.33	48.33	32.00	16.56
Novice	28.45	11.18	4.54	0.00

### Scenarios

In this study, the intermediate and novice surgical residents performed tasks in four scenarios in the following order: catch, reach, clean, and follow. The details of each scenario are given below.

#### Catch Scenario

**Figure 1.**
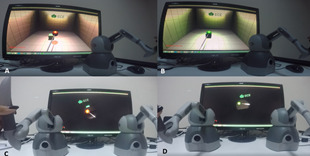
Catch Scenario

In this scenario, the participants were expected to catch a red ball that appeared at random locations on the screen in a room environment (Figure 1: A). To catch the ball, the participants had to use a surgical tool through a haptic device in an effective way. Their depth perception abilities played a great role in successfully completing each task. After catching this red ball, its color changes to green, then the participants were required to match it with the green cube (Figure 1: B). The participants were given 20 seconds to complete each of the 10 tasks included in this scenario.

The participants followed the same procedure first with the right hand, then with the left hand. As shown in Figure 1 (A and B), in this scenario there was no need to control the endoscope since the tasks were performed with a single hand using a surgical tool.

Another version of the scenario was designed for implementation with both hands. In this version, the participants were asked to control the source of light and camera (endoscope) with their left hand and the tool with their right hand. This version of the scenario required controlling the endoscope at an appropriate angle to determine the place of the ball and the box. Accordingly, the both-handed version of the scenario can be considered as a more challenging task than the single-handed version.

#### Reach Scenario

**Figure 2.**
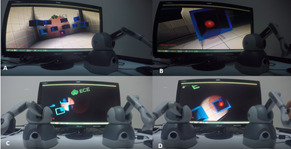
Reach Scenario

In this scenario, the participants were expected to reach a target location by identifying an appropriate angle. There were 10 red balls which randomly appeared in one of the blue boxes displayed on the screen (Figure 2: A). The participants were required to use the haptic device to home in on the red balls from the correct angle (Figure 2: B). Two versions of this scenario were prepared. The single-handed version can be considered as endoscope practice. In this scenario, they saw the environment from the camera perspective and performed the tasks by controlling the camera.

Once the correct angle was chosen, the ball disappeared. This process was undertaken 10 times, each with a time limit of 20 seconds. The both-handed version of this scenario was also more challenging since the participants had to maneuver the endoscope with their left hand and the operational tool with their right hand (Figure 2: C & D), requiring the effective use of both hands to accurately complete each task.

#### Clean Scenario

**Figure 3.**
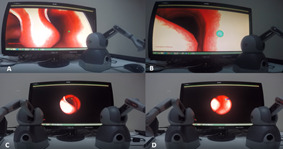
Clean Scenario

This scenario was also devised in two versions: single hand and both hands. In the first version, a nose model was created, and the participants were asked to clean the green balls that appeared in different locations inside this model. After cleaning each ball, another appeared in a different location (Figure 3: A & B). The participants were expected to carefully move to the location of each ball, catch it, and clean it. For each task, they were given 20 seconds as maximum time. If they could not perform the task in 20 seconds, this was recorded as a fail and the next trial started.

In the both-handed version, similar to the other scenarios, the participants were required to complete the similar tasks by controlling the endoscope with their left hand and the tool with their right hand (Figure 3: C & D).

#### Follow Scenario

**Figure 4.**
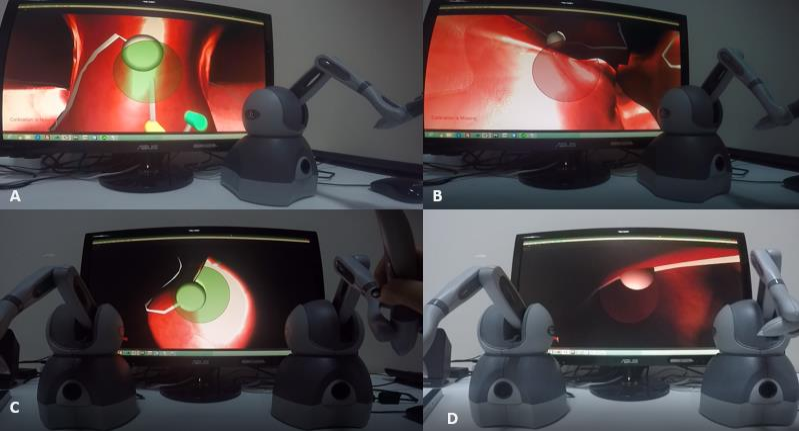
Follow Scenario

In this scenario, the participants were expected to follow a ball located at the beginning of a path (Figure 4: A). This path was presented in a human nose model environment. Using the haptic device to follow this ball to the end point of the path, the correct angle and distance had to be used.

In the single-handed version of the scenario, the participants followed the path through an endoscope view (Figure 4: A & B). However, in the both-handed version, they were required to control the endoscope with their left hand, and in a synchronized manner, they also need to maneuver the circle with their right hand to follow the path at a correct angle (Figure 4: C & D). In this scenario, the participants’ success was recorded at 10 equal distance points of the path.

### Apparatus

The pupil sizes of the participants were recorded using an eye tracker while they were performing computer-based simulated surgical tasks. The binocular eye-tracking technology was provided by The Eye Tribe Eye Tracker, which enables researchers to record pupil sizes of left and right eyes separately without a unit. Therefore, in this study pupil size variations were taken into consideration for understanding the differences between left- and right eye pupil sizes. In the literature, it is stated that pupil size differences can be significantly distinguished by an Eye Tribe eye tracker at different workload levels and the results of human factors research are promising (33).

### Procedure

The participants were seated in front of a monitor at a distance of 70 cm, and a verbal description of the procedure was given individually. The Eye Tribe Eye Tracker placed under the monitor recorded the pupil size differences of the participants while they were performing simulated surgical tasks. In order to prevent external factors participants’ head position and the experiment room luminance conditions were controlled. Furthermore, screen brightness and environment light were standardized. Also, ceiling lights were provided the same lighting conditions in the room. There were no feedback elements on the monitor that could cause to increase stress level of the participants (error-rate or score).

**Figure 5.**
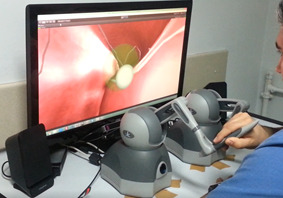
Procedure

The participants were instructed to look at the monitor continuously until the experiment ended (Figure 5). For ensuring the accuracy of the eye tracker, nine calibration points were presented. There are other methods which can be used for avoiding external effects on pupil sizes such as base line correction (34). This method was not applied in this study but it can be used to decrease the effect of unexpected pupil size fluctuations for improving the statistical power (34).

The participants performed the surgical scenarios under three hand conditions (right-hand, left-hand, and both hands) consecutively. The experiment started with the right-hand condition. After completion of all surgical scenarios, the participants were asked to complete them again with their left-hand. Lastly, they all followed the scenarios using both hands at the same time, with the right-hand controlling the haptic device as a surgical tool and the left-hand as an endoscope.

## Results

The statistical analyses were performed using SPSS for Windows software (version 23; IBM Corporation, New York, USA) at a 95% confidence level. Because of the normality assumptions are violated and sample size is 20 the parametric test techniques were not applied in this study. Therefore, non-parametric test techniques Mann Whitney test and Wilcoxon signed-ranks test were used (35). Mann Whitney; is a test technique used to compare two independent groups in terms of a quantitative variable and it is used for observing the differences between intermediate and novice surgeons and Wilcoxon signed-ranks test; is used to compare two paired samples when the assumptions for the parametric tests were not met (35).

**Figure 6.**
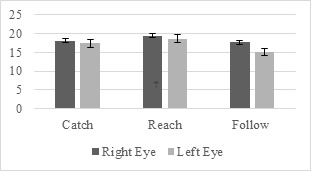
Left- and Right-Eye Pupil Size Differences under Right-Hand Condition for Novices

According to the Wilcoxon signed-ranks test results the left and right eye pupil size of the novice participants showed significant differences under right-hand condition (Figure 6).

For the catch scenario performed under the right-hand condition, the median right-eye pupil size rank (Mdn = 18.066) of the novices was significantly higher than that of the left-eye, Mdn = 17.412, Z = -2.936, p < 0.003.

Similarly, for the reach scenario, under the right-hand condition, the median right-eye pupil size rank (Mdn = 19.451) of the novices was significantly higher than that of the left-eye, Mdn = 18.627, Z = -2.949, p < 0.003.

For the follow scenario, under the right-hand condition, the median right-eye pupil size ranks (Mdn = 17.656) of the novice participants was significantly higher compared to the left-eye, Mdn = 15.081, Z = -2.949, p < 0.003.

**Figure 7.**
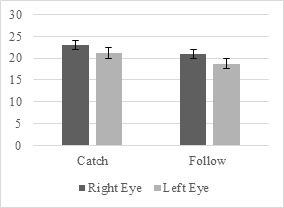
Left- and Right-Eye Pupil Size Differences under Both Hands Condition for Novices

Wilcoxon signed-ranks test results show that the left- and right-eye pupil size of the novice participants have significant differences under both hands condition (Figure 7).

Under the both hands condition in the catch scenario, results indicated that the median right-eye pupil size rank (Mdn = 23.010) of the novice level participants was significantly higher than that of their left-eye pupil size rank, Mdn = 21.170, Z = -2.669, p < 0.008.

Similarly, for the follow scenario, under the both hands condition, the median right-eye pupil size rank (Mdn = 20.965) of the novices was significantly higher than that of their left-eye, Mdn = 18.740, Z = - 2.694, p < 0.007.

**Figure 8.**
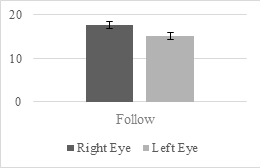
Left- and Right-Eye Pupil Size Differences under Right-Hand Condition for Intermediates

The left and right eye pupil size of the intermediate participants showed significant differences under right-hand condition based on Wilcoxon signed-ranks test results (Figure 8).

For the follow scenario under the right-hand condition, the median right-eye pupil size rank of the intermediates (Mdn = 17.656) was significantly higher compared to the left side, Mdn = 15.081, Z = -2.010, p < 0.003.

**Figure 9.**
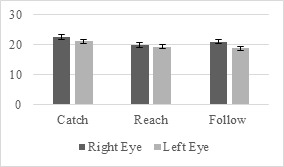
Left- and Right-Eye Pupil Size Differences under Both Hands Condition for Intermediates

The results of the Wilcoxon signed-ranks showed significant differences under both hands condition for the left- and right-eye pupil size of the intermediate participants (Figure 9).

For the catch scenario, under the both hands condition, the median right-eye pupil size rank (Mdn = 22.525) of the intermediates was significantly higher than that of left-eye, Mdn = 21.107, Z = -2.092, p < 0.036.

For the reach scenario under the both hands condition, the median right-eye pupil size rank of the intermediates (Mdn = 19.861) was significantly higher compared to the left side, Mdn = 19.286, Z = -2.629, p < 0.009.

Similarly, when performing the follow scenario tasks under the same condition, the median right-eye pupil size ranks of the intermediates (Mdn = 20.965) was significantly higher than that of their left-eye, Mdn = 18.740, Z = -2.751, p < 0.006.

Since the normality assumptions were not satisfied and the sample size was 20, the Mann Whitney non-parametric test technique (35) was used compare the two independent groups; i.e., intermediate and novice surgeons in terms of pupil sizes.

**Figure 10.**
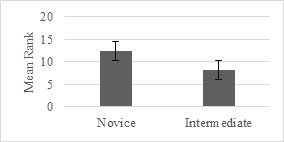
Right-Eye Pupil Size Differences between Novice and Intermediates under Right-Hand Condition in Catch Scenario

There was a statistically significant difference in the pupil sizes between the novices and intermediates. As shown in Figure 10, the right-eye pupil sizes of the novice surgeons were significantly larger than those of the intermediate surgeons (U = 22, p < .05) for the catch scenario under the right-hand condition. However, for the clean scenario, no significant differences were found under any of the three hand conditions. Similarly, the left-hand condition did not result in a significant difference between the two groups in any of the scenarios.

## Discussion

In this study, four different scenarios, each involving 10 tasks were used. All participants were right-handed, and all scenarios were performed under the right-hand, left-hand and both hands conditions consecutively.

Blumenfeld (20) reported that the left- and right-eye pupil sizes were equal under normal conditions, but there were differences between the two under different conditions. Similarly, our results indicated some significant differences between the right and left pupil sizes of the intermediate and novice surgical residents.

For instance, under the both hands condition, in the catch, reach and follow scenarios, the right pupil size of the novices was significantly larger than their left pupil size. For the intermediate surgical residents, this situation was similar for the both hands condition, except the reach scenario, in which no significant change was found.

Under the right-hand condition, the intermediate groups’ right pupil sizes were significantly large compared to the left-eye in the catch, reach and follow scenarios. The right pupil sizes of the novice group were also significantly larger than their left pupil sizes for the right-hands condition in the follow scenario. For the novice group, no significant difference was found for the remaining scenarios under the right-hand condition.

In some cases, no significant difference was found between the left- and right-eye pupil sizes: all conditions in the clean scenario and the left-hand condition in all scenarios.

The results of this study showed that under different conditions the left and right pupil sizes of the surgical residents may significantly differ when performing different tasks. However, it should be noted that our participants were all right-handed, which was the reason why the right pupil was generally significantly higher than the left pupil during the completion of the tasks. If similar tasks were performed with left-handed surgical residents, we consider that the opposite results would be obtained.

In one case, the catch scenario, the right-hand condition resulted in a significant difference between the intermediate and novice participants, with the latter having significantly larger right pupils than the former. This result indicates that experience needs to be carefully considered to provide a better understanding of the pupil size differences between the left and right sides. Our results are parallel to those reported by Wahn, Ferris (27), confirming pupil size asymmetries. However, we also found that in some cases, the individuals’ right pupil was larger than their left pupil. Another noteworthy finding was that there were no significant differences for the tasks performed under the left-hand condition, which was attributed to all participants being right-handed.

Lastly, for the clean scenario, which was considered to the hardest of all four, no significant difference was observed. Hence, the effect of the difficulty level of the scenario and its appropriateness with the skill level of the participants need to be further investigated.

## Conclusion

This study revealed certain differences between the left and right pupil sizes of the novice and intermediate surgical residents. By considering these differences, further eye-movement research can offer a deeper understanding of human behaviors. The experience levels, handedness (left-handed or right-handed), and the conditions in which tasks are performed need to be carefully investigated by analyzing the asymmetries in the right and left pupil sizes. Also, to prevent the effect of unexpected pupil size fluctuations and improve the statistical power of the analysis baseline correction method can be used (34).

Because of the four scenarios were not performed in randomized order there might be an order effect. Even this order affect, the results show significant differences between right and left-pupil sizes. Accordingly, this order affect can be considered as acceptable for this study. Also, as the tasks under the both hands condition were performed after the conditions of left and right hands which can be considered as fatigue increasing the fluctuations of the pupil size and possibly making it larger. Therefore, in future studies, this order effect can be controlled. Additionally, as the number of surgeons in the endo-neurosurgery is very limited, this study could be conducted with a limited number of surgeons. For the future studies, the hypothesis of this study can be tested with a bigger group of participants.

## Ethics and Conflict of Interest

The author(s) declare(s) that the contents of the article are in agreement with the ethics described in http://biblio.unibe.ch/portale/elibrary/BOP/jemr/ethics.html and that there is no conflict of interest regarding the publication of this paper.
